# R-Silsesquioxane-Based Network Polymers by Fluoride Catalyzed Synthesis: An Investigation of Cross-Linker Structure and Its Influence on Porosity

**DOI:** 10.3390/ma13081849

**Published:** 2020-04-15

**Authors:** Nai-hsuan Hu, Joseph C. Furgal

**Affiliations:** Department of Chemistry and Center for Photochemical Sciences, Bowling Green State University, Bowling Green, OH 43403, USA; naihsuh@bgsu.edu

**Keywords:** silsesquioxanes, sol-gel, network polymers, porosity, fluoride catalysis, POSS, aerogels, alkoxysilanes

## Abstract

Silsesquioxane-based networks are an important class of materials that have many applications where high thermal/oxidative stability and porosity are needed simultaneously. However, there is a great desire to be able to design these materials for specialized applications in environmental remediation and medicine. To do so requires a simple synthesis method to make materials with expanded functionalities. In this article, we explore the synthesis of R-silsesquioxane-based porous networks by fluoride catalysis containing methyl, phenyl and vinyl corners (R-Si(OEt)_3_) combined with four different bis-triethoxysilyl cross-linkers (ethyl, ethylene, acetylene and hexyl). Synthesized materials were then analyzed for their porosity, surface area, thermal stability and general structure. We found that when a specified cage corner (i.e., methyl) is compared across all cross-linkers in two different solvent systems (dichloromethane and acetonitrile), pore size distributions are consistent with cross-linker length, pore sizes tended to be larger and π-bond-containing cross-linkers reduced overall microporosity. Changing to larger cage corners for each of the cross-linkers tended to show decreases in overall surface area, except when both corners and cross-linkers contained π-bonds. These studies will enable further understanding of post-synthesis modifiable silsesquioxane networks.

## 1. Introduction

Sol-gel reactions of R- and bis-R-alkoxysilanes have been used over the years to synthesize a wide variety of highly porous materials containing hybrid inorganic/organic moieties [1,2,3,4,5,6]. Although historically these types of porous materials have focused on using bridged alkoxysilanes synthesized by acid or base catalysis in a random structural formation, there are many advances in controlling the reactivity that give more focused pore sizes and overall reaction control [7,8,9,10,11,12,13,14]. There have been a few strategies implemented for this, including extensive processing and templation methodologies to maximize porosity and pore-structure stability [10,12,15,16,17,18,19,20,21]. The advancements and mechanistic understanding of fluoride catalysis (non-stoichiometric) as a technique to synthesize these types of structures has enabled vast improvements in these materials from a structural and synthetic control standpoint [22,23]. For example, Corma et al. have found good synthetic control with fluoride catalysis methods and showing bridging moiety control of porosity [23]. These improvements are due to the rapid equilibration processes that occur, which allow for the facile generation of more uniform porosity in materials as well as controlled swellability [13,15,24,25,26,27,28,29].

Recently, we reported on the tetrabutylammonium fluoride-(TBAF) catalyzed synthesis of methyl-silsesquioxane-based ethane bridged network materials, where we explored the importance of reaction solvent and water content in modifying the overall pore size distributions of a single cross-linker system [29]. Though many reports speculate that the pore sizes obtained in sol-gel reactions are directly related to the bridging groups used, especially for rigid spacers [2,26,30,31,32], we found that multiple pore size distributions (0.5 to 100 nm) could be obtained by simply changing the reaction solvent for the same bridge. For example, dichloromethane (DCM) favored micropores on the order of 1.2 nm and gel particles, while changing the reaction solvent to acetonitrile (ACN) gave pores centered around 3 nm and favored global gelation of the entire system. All the synthesized materials favored non-polar solvents as expected for organogels based on networked silsesquioxanes.

Though high porosity materials with static or non-functionalizable pores are useful in many applications [33,34,35,36], it is often desirable to impart additional functionality to these materials for many specialized uses. such as in biology or environmental remediation. as capture and release agents [2,17,37,38,39]. These functionalities may include hydrophilic groups, amino-acids and reversible chemistries such as “click” or complexation ligands [40,41,42,43,44]. Many researchers have been working on methods to synthesize active silsesquioxane-based networks, albeit primarily through preformed cage methods. In terms of preformed cage systems, Ervithayasuporn et al. have developed a recyclable methacrylate-POSS (polyhedral oligomeric silsesquioxanes) porous network which can efficiently bind Pd and acts as a catalyst for alcohol oxidation in the presence of water [45]. Shimojima et al. developed a photo-responsive azobenzene-based porous network using hydrosilylation chemistries [46], and Liu et al. have used Heck cross-coupling to synthesize porous POSS with surface areas up to 600 m^2^ g^−1^ and used them for water purification by removal of lead [17]. In regards to active in-situ-formed networks more similar to this work, Gan et al. have developed a series of luminescent networks materials using pyridinium-based cross-linkers [47], and Moreau et al. have synthesized a series of chiral-bridged porous materials that impart helical structure during the sol-gel process [12].

While the methods above describe the most desirable methods to incorporate specific functionalities before synthesis, there are often challenges with monomer purification or compatibility with the other components during synthesis (i.e., phase separation). Therefore, the incorporation of R-alkoxysilanes or bridging groups with the ability of post-synthesis modification is sought after and may include reactive systems such as ethylene, vinyl, alkynyl, phenyl, azido, amino or other substitutable groups [27,38,48,49,50,51]. One of the challenges with incorporating functionalizable R-substituents within the structure is that many of these components have large sizes, π-stacking or hydrogen bonding that can hinder the accessibility of pores to analytes or modification reactions. In this case, the choice of synthesis technique and/or post-synthesis-processing methods is very important to keep pores open for future reactions. An additional goal is to not only impart later functionalization, but to be able to control pore sizes to include or exclude different species of interest, something that is still difficult to achieve.

In this study we expand on our previous findings on the importance of solvent in controlling pore size distributions in ethyl-bridged methylsilsesquioxane networks. We apply our knowledge of those simple systems to determine how the incorporation of other silsesquioxane building blocks such as phenyl/vinyl-silyl corner groups and cross-linker length and rigidity affect the properties of fluoride-catalyst-synthesized post-synthesis modifiable porous networks.

## 2. Materials and Methods

Materials: Methyltriethoxysilane (CH_3_Si(OEt)_3_, MeSi(OEt)_3_), vinyltriethoxysilane (CH_2_CHSi(OEt)_3_, vinylSi(OEt)_3_), phenyltriethoxy-silane (C_6_H_5_Si(OEt)_3_, PhSi(OEt)_3_), 1,2-bis(triethoxysilyl)ethane ((EtO)_3_SiCH_2_CH_2_Si(OEt)_3_, BTSE), 1,2-bis(triethoxysilyl)ethylene ((EtO)_3_SiCHCHSi(OEEt)_3_, BTSEE), 1,6-bis(triethoxysilyl)hexane ((EtO)_3_Si(CH_2_)_6_Si(OEt)_3_, BTSH) and 1,2-bis(triethoxysilyl)acetylene ((EtO)_3_SiCCSi(OEt)_3_, BTSA) were purchased from Gelest, Inc., Morrisville, Pennsylvania, PA, USA. Acetonitrile (ACN) and dichloromethane (DCM) were purchased from Millipore Inc. Saint Louis, Missouri, MO, USA. Tetrabutylammonium fluoride (TBAF, 1.0 M in THF) was purchased from Acros Organics, Morris Plains, New Jersey, NJ, USA. Other chemicals and reagents as noted in the text were used as received.

General condensation reactions: 6.25 mmol R-Silsesquioxane-Based porous networks by fluoride catalysis containing methyl, phenyl and vinyl corners (R-Si(OEt)_3)_, 1.75 mmol of cross-linker, and 0.75 mL H_2_O were mixed in a 500 mL round-bottom flask. The mixture was then stirred by a magnetic bar for 5 min with 200 mL of reagent grade acetonitrile (ACN) or dichloromethane (DCM) as solvent. Tetrabutylammonium fluoride (TBAF) was then added to the mixture as catalyst. After 24 h of reaction, the reaction mixture was filtered if precipitate was formed. Remaining gel in the filter was then rinsed by corresponding solvent to remove the catalyst. Reactions retained without precipitation (i.e., sol- or global gel) for 24 h were transferred to a 500 mL beaker, then the mixture was dried under ambient. Gels from both methods were dried under high vacuum for 24 h, ground to powder by mortar and pestle, then dried again under high vacuum to ensure the absence of solvent. These reaction conditions were used for the general synthesis of all materials studied in this manuscript. Each sample was synthesized three times, with surface area and full characterization conducted on the 2nd samples for consistency.

Analytical methods: Fourier–transform infrared spectroscopy (FTIR). All of the spectra were obtained from Thermo Scientific Nicolet iS5 Fourier Transform Infrared Spectrometer (Waltham, Massachusetts, MA, USA). Grounded samples were placed directly onto a ZnSe crystal, then scanned from 4000 to 400 cm^−1^ for 32 scans with 0.121 cm^−1^ resolution. Attenuated total reflection (ATR) method was applied to the analysis.

Specific surface area (SSA) and porosity analyses: A Micromeritics 3FLEX surface and catalyst characterization analyzer (Micromeritics Inc., Norcross, GA, USA) was used to analyze the surface area and porosity of all samples [29,52]. Samples were degassed at room temperature for 2 h with an N_2_ flush. The surface area was calculated by Brunauer–Emmet–Teller (BET) method. Pore volume and pore size distributions were calculated by density functional theory (DFT) methods. The measurements were carried out at −196 °C (77 K) while collecting N_2_ adsorption and desorption isotherms. The samples were first evacuated to 0.0001 mmHg then gradually dosed with N_2_ gas until reaching saturated pressure (760 mmHg). P/Po of the first adsorption point were calculated as single point surface area. The samples were then gradually evacuated to P/Po points as set up to obtain desorption graph. In this paper, the mesopore data were determined by the multipoint method using 20 data points with 0.050 (p/p0) relative pressure increment starting from a relative pressure 0.015 (p/p0). The data were calculated by DFT method to obtain pore size distributions. Absorption and desorption isotherms are given in supporting information for all samples (Figure S1).

Thermalgravimetric analysis (TGA): ceramic yields and T_5%_ were measured by a TGA−50 (TA Instruments, Inc., New Castle, DE, USA) instrument. Ground samples of 10–20 mg of material were put into an alumina pan. The sample was heated under air (60 mL/min) from room temperature to 1000 °C at a rate of 10 °C/min.

Solid state nuclear magnetic resonance: solid state ^29^Si NMR was measured by a Bruker Avance III NMR spectrometer (Bruker, Inc., Billerica, MA, USA). A multinuclear double resonance CP-MAS probe was tuned to 119.23 MHz with a 4 mm top-loading rotor. Ground samples were measured for 256 scans with a 20 to −160 ppm spectral window and 7 s of decay time. Experiments were run with a tightly packed zirconia spinner and spin rate of 9 kHz.

## 3. Results and Discussion

**Scheme 1:**
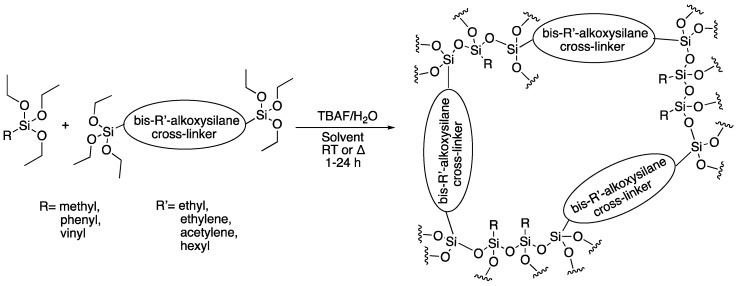
Reaction to form silsesquioxane networks using fluoride-catalyzed synthesis. TBAF: tetrabutylammonium fluoride. *RT = room temperature.

Cross-linkers with various electron density, rigidity and length were reacted with R-Si(OEt)_3_ to determine how they impact structure and properties such as surface area, porosity, reactivity and sol-gel reaction completion, where R = Me, phenyl, vinyl (Scheme 1). While these types of cross-linkers have been explored in great deal in the literature, combinations of these with various corners under reversible fluoride conditions are still underexplored. Sol-gel reaction efficiencies were measured by TGA, based on analysis of post-curing wt. % drops at ~200 °C. We compared three cross-linkers against BTSE systems: [29] bis-triethoxysilylhexane (BTSH), bis-triethoxysilylethene (BTSEE), and bis-triethoxysilylacetylene (BTSA). We used a ratio of 1:0.36 of R-Si(OEt)_3_ to cross-linker for our standard conditions, as they have been found to offer the highest surface areas and best overall reaction completion [29]. We looked at reactions in two solvents, one of moderate polarity and low water miscibility, DCM, and one of high polarity and water miscibility, ACN. These solvents were chosen because of results from previous BTSE studies, which gave different mechanisms of reactivity, whereby DCM leads to precipitated gel particles and ACN gives global gelation in water-rich systems. In most sol-gel type processes, post-curing is necessary to reach reaction completion. However, in these systems using either DCM or ACN no post-curing was done in order to better compare results across linkers. This decision was reinforced by a tendency for reaction completion to be generally quite high and we wanted to limit the oxidation of π-bonds. All materials when dried out gave a white or translucent appearance and were amorphous in structure. The FTIRs for all samples in this series are given in Figure S2.

### 3.1. Cross-Linkers

**Figure 1:**
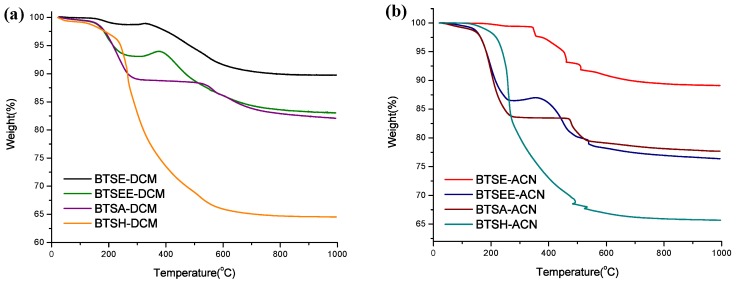
Thermal Gravimetric Analysis (TGA) graphs (in air) of materials from reactions with different cross-linkers in (**a**) dichloromethane and (**b**) acetonitrile, note BTSEE shows oxidation of the double bond at 400 °C.

**Figure 2:**
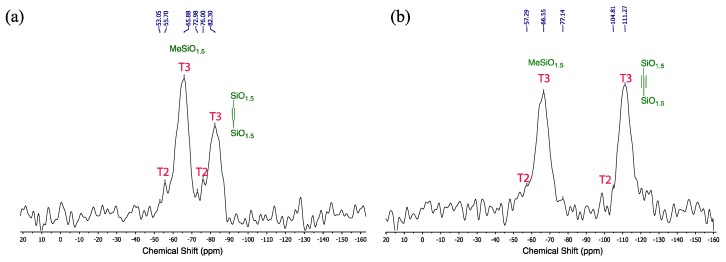
Solid state ^29^Si nuclear magnetic resonance (NMR) of (**a**) Me-BTSEE-based network polymer from DCM showing unreacted ethoxy shoulders (T_2_) at −55 and −76 ppm and main peak for T_3_ at −65 and −85, respectively, (**b**) Me-BTSA-based network polymer from DCM showing unreacted ethoxy shoulders (T_2_) at −67 and −104 ppm and main peak for T_3_ at −66 and −111, respectively.

We started our study with methyl corner groups and exchanging out BTSE for each of the above cross-linkers (BTSEE, BTSA and BTSH) using standard conditions in DCM and ACN. We found that any of the above cross-linkers resulted in approximately half the surface area or less, regardless of solvent versus BTSE on its own (Table 1). Some of this decline can be attributed to low reaction completion efficiencies. Weight drops occurring below 200 °C imply the presence of unreacted ethoxy/hydroxy groups in the structure. BTSEE and BTSA linkers both showed an especially prevalent drop of up to 15%, which leads to lower ceramic yields than expected in the TGA for these samples (Figure 1). This is further confirmed by solid state ^29^Si NMR (Figure 2 and Figure S3), which shows significant shoulders corresponding to ethoxy groups in both of these samples [53]. Solid state ^29^Si NMR additionally shows that BTSEE and BTSA are effectively incorporated into the network by their distinctive positions at −82 and −111 ppm, respectively. In BTSH samples, the breadth of the solid state ^29^Si NMR signal masks the cross-linkers and it cannot be deciphered from that of methyl-Si corner bonding.

Reactions with cross-linkers BTSEE and BTSA in DCM show precipitation within 1 h of adding catalyst to the reaction, with specific surface area (SSA) trending similarly with values of 556 and 454 m^2^ g^−1^. This decline in SSA for BTSEE and BTSA can most likely be attributed to difficulties in the system reaching reaction completion. Incomplete networks contain large amounts of pore-blocking ethoxy/hydroxy groups. This is evidenced by “post-curing” drops in the TGA below 200 °C (Figure 1a). For example, with BTSA and BTSEE, though pore size distribution patterns are similar to reactions with BTSE, they show 10% and 7% drops in this “post-curing” region of the TGA, which result in a loss of ~0.2 cm^3^/g of cumulative pore volume, equivalent to half the BTSE SSA. In addition, materials with BTSEE in both solvents show oxidation peaks at around 375 °C, this indicates that the ethylene π-bonds remain chemically active for further functionalization [54,55].

In contrast, BTSH shows a significant drop-off in SSA in DCM to 112 m^2^ g^−1^ (Table 1), which is most likely attributed to a significant increase in pore size distribution and the increased flexibility and length of the linker. BTSH is also the only DCM sample to exhibit extensive hysteresis between adsorption and desorption isotherms in BET analysis, suggesting difficulty in removing N_2_ (Figure S1). Other samples show very little hysteresis. For reactions with BTSH, precipitation was not observed within 24 h and the reaction mixture was air-dried in a beaker resulting in global gelation occurring during the drying process. The flexibility of this linker gives a higher solubility for intermediates, but also a higher propensity for pore collapse [56]. This leads to lower surface areas being obtained when compared to the more rigid BTSEE and BTSA linkers.

In ACN samples there is a significant trend toward decreasing surface areas with increasing rigidity and electron density of the cross-linker (Table 1, Figure 1b). This difference between DCM and ACN is most likely attributed to the solubility variations of intermediate fragments during synthesis [29]. Going from BTSE to BTSEE to BTSA shows a narrowing of pore size distributions, but a trend towards mesopores versus DCM samples. Reactions with BTSA only show half the surface area (212 m^2^ g^−1^) compared with BTSEE (423 m^2^ g^−1^). This suggests reactions with these two different linkers have distinctive equilibration methods in forming intermediate structures and thus result in dissimilar pore size distributions. All samples synthesized in ACN show primarily type II-hysteresis loops that suggest a rigid structure of xerogels, and some difficulty in gas removal (Figure S1). Reactions with BTSH in ACN also show no precipitation within 24 h and are held in solution until concentrated. These materials resulted in pore size distributions more closely resembling BTSE in ACN. This contrasts many studies on the influence of cross-linker length on pore size in bis-alkoxysilyl-R systems, which suggest that increasing cross-linker length increases pore size, however rigidity and catalyst also play a role [56]. This effect will be the topic of future investigation.

**Figure 3:**
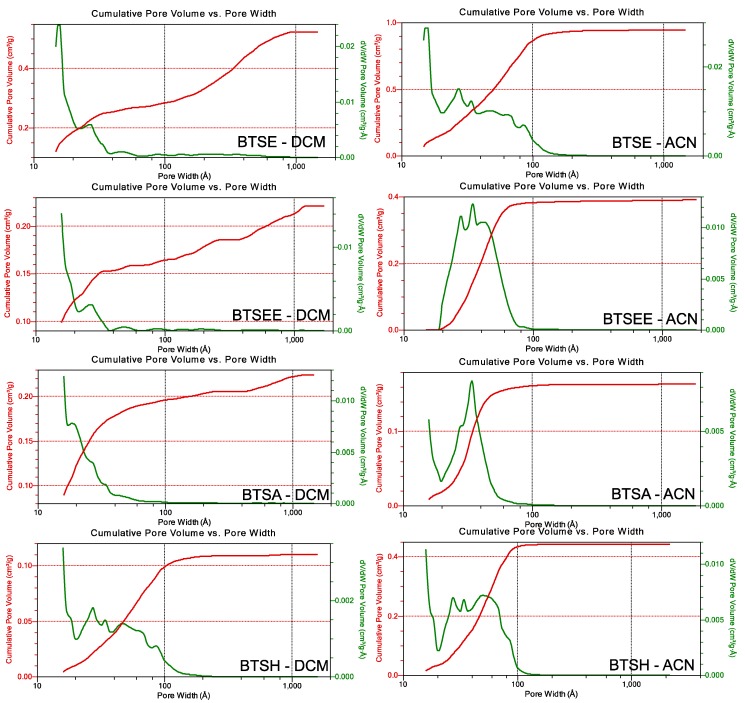
Density functional theory (DFT) method pore size distributions and cumulative pore volume from Brunauer–Emmet–Teller (BET) method measurement for Me-BTSE, Me-BTSEE, Me-BTSA and Me-BTSH in both DCM and ACN.

There is considerable difference in the pore size distributions between DCM and ACN solvent systems. For all samples except for BTSH, the DCM solvent system results in overall smaller pores than that for the can-based reactions (Figure 3). This is expected based on previous studies and shows the importance of solvent choice when making silsesquioxane networks. The lower reaction efficiency of the shorter, more rigid cross-linkers is most likely due to steric hindrance from the growing partial cages nearby. This is more significant for the shortest spacer (BTSA) than even for BTSEE. One of the interesting anomalies is that the pore distribution for the BTSEE and BTSA samples in ACN show little microporosity in the distribution compared with BTSE-based materials, and their current studies in DCM; instead they contain mostly mesopores. It would be expected that these shorter and more rigid cross-linkers would be less influenced by solvent effects, but it appears that there must be a strong interaction between ACN and the cross-linkers, in which ACN could be associating itself with the cross-linker π-bonds and influencing the pore structure. Alternatively, there could be enough unreacted ethoxy/hydroxy groups in the structure to increase the pore sizes which would also correlate with the decreased SSA as previously mentioned. This would conflict with the FTIR analysis where there does not appear to be an abundance of hydroxy groups.

### 3.2. Corner-Groups

**Figure 4:**
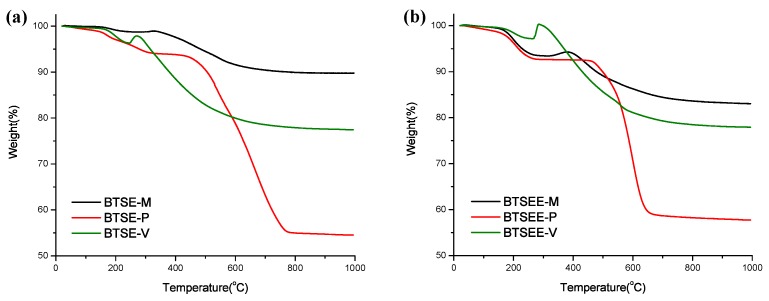

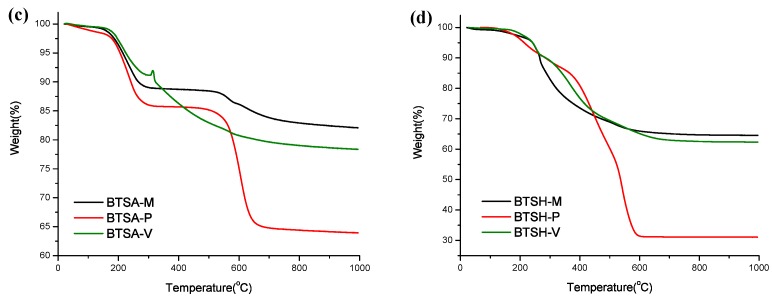
TGA graphs of materials from reactions with three corner-silanes and (**a**) BTSE, (**b**) BTSEE, (**c**) BTSA, (**d**) BTSH from DCM. BTSE: 1,2-bis(triethoxysilyl)ethane; BTSEE: 1,2-bis(triethoxysilyl)ethylene; BTSA: 1,2-bis(triethoxysilyl)acetylene; BTSH: 1,6-bis(triethoxysilyl)hexane; DCM: dichloromethane; ACN: acetonitrile.

In addition to the above study comparing different cross-linkers and their influence on porosity, we were interested in changing out the methyl-based silsesquioxane corners for two other common R-groups, which could be further functionalized by post-synthesis modification. These groups have the potential for different steric, electronic and polarity interactions [57]. Though the use of methyl R-groups is very attractive and should theoretically maximize the porosity in these systems, phenyl and vinyl R-group replacements were explored and their influence on structure-property relationships in DCM were determined to make a clear comparison based only on functional groups. As expected, we observed significant reductions in the ceramic yield and surface area when switching to larger spatially consuming R-groups (Table 2, Figure 4). The decrease in SSA is most likely due to decreasing pore accessibility. Note that all reactions with BTSH cross-linkers show good conversions in the sol-gel process by lack of mass loss at ~200 °C, but show the lowest overall thermal stability with decomposition starting to take place at ~300 °C (in air). It is also interesting that BTSH networks did not form gel particles upon reaction in any experiments before concentration. Once the other cross-linker systems have been “post-cured” in TGA to remove residual ethoxy/hydroxy groups they show thermal stabilities of ~400 °C. The FTIR for all samples in this series are given in Figure S4.

Reactions with phenyl corners tend to give the best network formation with BTSEE and BTSA. These have substantially more cumulative pore volume (>0.2 cm^3^ g^−1^) and SSA than their BTSE and BTSH counterparts (~0 m^2^ g^−1^ SSA), which seem to react poorly or have extensive pore collapse, and have cumulative pore volumes less than 0.001 cm^3^ g^−1^ (Table 2 and Figure S5). Furthermore, reactions with BTSEE and BTSA tend to favor microporosity versus BTSE and BTSH, which favor mesoporosity, albeit all systems are relatively disperse in porosity. The reasoning for this distinction likely lies in the ability for π-bond overlap between the BTSEE and BTSA cross-linkers with the phenyl corners, which can enable an almost templated alignment for reactions to occur. The lower cumulative pore volume of the BTSA (0.2 cm^3^ g^−1^) systems correspond closely to the ~15% drop in the TGA curve from incomplete reactions. So even with the possibility of π-bond overlap selectively enabling smaller pore sizes, they do little to aid in reaction completion [58]. An alternative explanation for this phenomenon would be based on cross-linker rigidity, which was discussed in great detail by Shea and Loy [26], as well as Corma et al. [23]. BTSA and BTSEE offer far more rigidity than those of BTSE and BTSH and by locking the system together more tightly the pores become more accessible in the cross-linkers with π-bonds. This theory is supported by the SSA’s found for each system, with BTSE and BTSH offering no surface area (i.e., non-accessible pores), while both BTSA and BTSEE are above 200 m^2^ g^−1^.

Networked materials with vinyl groups on the corners tend to show similar trends to that as phenyl, but are less hindering to overall sol-gel reaction efficiency as evidenced by TGA (Figure 4) and SSA in Table 2. Vinyl groups from vinylSi(OEt)_3_ stay chemically active in the material and show a slight oxidation peak at around 300 °C with BTSE, BTSEE and BTSA. This is due to the reactivity of the vinyl groups when heated in the presence of oxygen [49,50]. For each of the vinyl corners, except for reactions with BTSH, there was SSA of 455, 492 and 553 m^2^ g^−1^ respectively for BTSA, BTSEE and BTSE, which are too similar to make major inferences about them. While for BTSE the SSA is about half that for methyl, the good compatibility and higher rigidity of BTSA and BTSEE samples with vinyl offer very similar surface areas to their methyl counterparts and in the case of BTSA, it is slightly higher. In reactions with BTSA, BTSEE and BTSE, similar pore distributions are achieved, primarily in the micropore size domain, while those with BTSH have a very broad pore distribution spanning from ~5 to >100 nm (Figure S5). Materials with BTSH and vinyl did not show any significant surface area.

One of the interesting effects of the addition of cross-linkers and corner groups that contain π-bonds is that their slight electron-withdrawing nature should allow more efficient attack of fluoride on silicon and thus make the reactions more dynamic in nature. Laine et al. have described multiple instances where addition of π-containing silsesquioxane systems enables better rearrangement of POSS cages [59,60]. So it is quite surprising that we see far less reaction completion for the π-bond-containing structures and as such sterics and rigidity of the components must have a stronger influence than electronics on the material formation.

## 4. Conclusions

We have found that cross-linker and corner monomer choice largely affect surface area, reaction completion efficiency and pore size distribution for fluoride-catalyzed network polymer formation. Rigid cross-linkers such as BTSEE or BTSA tend to decrease surface area in both DCM and ACN, and give a narrower pore size distribution in ACN than DCM. Longer and more flexible crosslinkers like BTSH cause products to remain in solution indefinitely and these reactions must be concentrated to dryness in order to get solid or gel precipitate, often leading to large pore distributions and low SSA. There is no evidence of a relevant trend in pore size distributions related to cross-linker lengths and rigidities, with BTSEE and BTSA giving overall larger pores than those observed for BTSE. This suggests that the addition of corner units complemented with cross-linkers alters the structure substantially over using cross-linkers alone. Reactions with exchanged corners from Me to Ph or vinyl tend to show significant decreases in surface area with BTSE and BTSH, however due to favorable interactions between the Ph and vinyl systems with π-containing cross-linkers (BTSEE and BTSA) these systems showed approximately the same or higher SSA relative to BTSE. These results further our understanding of fluoride-catalyzed silsesquioxane network formation studies and give increased evidence for the importance of solvent choice in determining material properties. In addition, we discovered that both vinyl groups in corner groups and π-bonds in BTSEE are still chemically active after polymerization. This insight offers the ability to incorporate reactive moieties as corner groups and cross-linkers. For example, amino acids or thiol ether groups can be incorporated in the material to improve hydrophilicity. This reaction methodology gives great potential of post-synthesis modifications for applications ranging from selective capture of environmental contaminants such as lead, phosphorous or oil, to separation materials for drugs and oil/water mixtures.

## Figures and Tables

**Table 1:** Specific surface areas and thermalgravimetric analysis (TGA) decomposition and ceramic yield of materials from reactions with different cross-linkers in dichloromethane and acetonitrile.

Cross-Linker	Solvent	P = Particle N = No Gel	Surface Area (m^2^ g^−1^)	Temperature at 5% Mass Loss (*T*_5%_)	Residue at 1000 °C (Ceramic Yield)
BTSE					
	DCM	P	1076	361.3 °C	86.2%
	ACN	P	1022	451.6 °C	89.2%
BTSEE					
	DCM	P	556	251.2 °C	83.1%
	ACN	P	423	183.4 °C	76.5%
BTSA					
	DCM	P	454	214.7 °C	82.2%
	ACN	P	212	182.9 °C	77.7%
BTSH					
	DCM	N	112	241.4 °C	64.6%
	ACN	N	414	239.2 °C	65.7%

BTSE: 1,2-bis(triethoxysilyl)ethane; BTSEE: 1,2-bis(triethoxysilyl)ethylene; BTSA: 1,2-bis(triethoxysilyl)acetylene; BTSH: 1,6-bis(triethoxysilyl)hexane; DCM: dichloromethane; ACN: acetonitrile.

**Table 2:** Surface areas of materials from reactions with different corner-silanes and cross-linkers in DCM.

Cross-Linker	Corner Silane	P = Particle N = No Gel	Surface Area (m^2^ g^−1^)	Temperature at 5% Mass Loss (*T*_5%_)	Residue at 1000 °C (Ceramic Yield)
BTSE					
	MeSi(OEt)_3_ (methyl)	P	1076	483.7 °C	89.7%
	PhSi(OEt)_3_ (phenyl)	P	1	278.9 °C	54.6%
	VinylSi(OEt)_3_ (vinyl)	P	553	315.2 °C	77.5%
BTSEE					
	MeSi(OEt)_3_	P	556	229.1 °C	83.1%
	PhSi(OEt)_3_	P	245	216.8 °C	57.8%
	VinylSi(OEt)_3_	P	455	369.3 °C	78.0%
BTSA					
	MeSi(OEt)_3_	P	454	214.7 °C	82.2%
	PhSi(OEt)_3_	P	316	205.7 °C	64.0%
	VinylSi(OEt)_3_	P	492	225.0 °C	78.5%
BTSH					
	MeSi(OEt)_3_	N	112	241.4 °C	64.6%
	PhSi(OEt)_3_	N	-	213.5 °C	31.1%
	VinylSi(OEt)_3_	N	-	242.0 °C	62.4%

## Supplementary Materials

The following are available online at https://www.mdpi.com/1996-1944/13/8/1849/s1. Figure S1: (a) Isotherm graphs from reactions with different cross-linkers in dichloromethane (DCM) and acetonitrile (ACN), Plus (light red) = adsorption, circle (dark red = desorption). (b) Isotherm graphs of materials from reactions with different corner-silanes and cross-linkers, P = phenyl, V = vinyl, M = methyl from DCM, Plus (light red) = adsorption, circle (dark red = desorption), Figure S2: IR spectra of materials from reactions with different cross-linkers in dichloromethane (DCM) and acetonitrile (ACN), Figure S3: ^29^Si NMR of methyl silsesquioxane based network polymer with hexyl spacer made in DCM. Note there are not extensive shoulders or side peaks indicating near complete reaction, Figure S4: IR spectra of materials from reactions with different corner-silanes and cross-linkers, P = phenyl, V = vinyl, M = methyl from DCM, Figure S5. DFT pore size distribution plots and cumulative pore volume of materials from reactions with different corner-silanes and cross-linkers, P = phenyl, V = vinyl, M = methyl from DCM.
