# Supplementary material for: R-Silsesquioxane-Based Network Polymers by Fluoride Catalyzed Synthesis: An Investigation of Cross-Linker Structure and Its Influence on Porosity
Source: Materials (Basel). 2020 Apr 15;13(8):1849. doi: 10.3390/ma13081849 (PMC7215510; doi:10.3390/ma13081849)
Supplement: Supplementary file 1 [file materials-13-01849-s001.pdf]

materials

Supplementary

R-Silsesquioxane-Based Network Polymers by Fluoride Catalyzed Synthesis: An Investigation of Cross-linker Structure and its Influence on Porosity

Nai-hsuan Hu and Joseph C. Furgal *

Department of Chemistry and Center for Photochemical Sciences, Bowling Green State University, Bowling Green, OH 43403, USA; naihsuh@bgsu.edu

* Correspondence: furgalj@bgsu.edu

Page	Content
S1	Table of contents
S2	Isotherm graphs of materials from reactions with different corner-groups and cross-linkers.
S4	IR spectra of materials from reactions with different cross-linkers in dichloromethane (DCM) and acetonitrile (ACN).
S5	^{29}Si NMR of Me-BTSH network in DCM.
S6	IR graphs of materials from reactions with different corner-groups and cross-linkers.
S8	Pore size distribution graphs of materials from reactions with different corner-groups and cross-linkers.

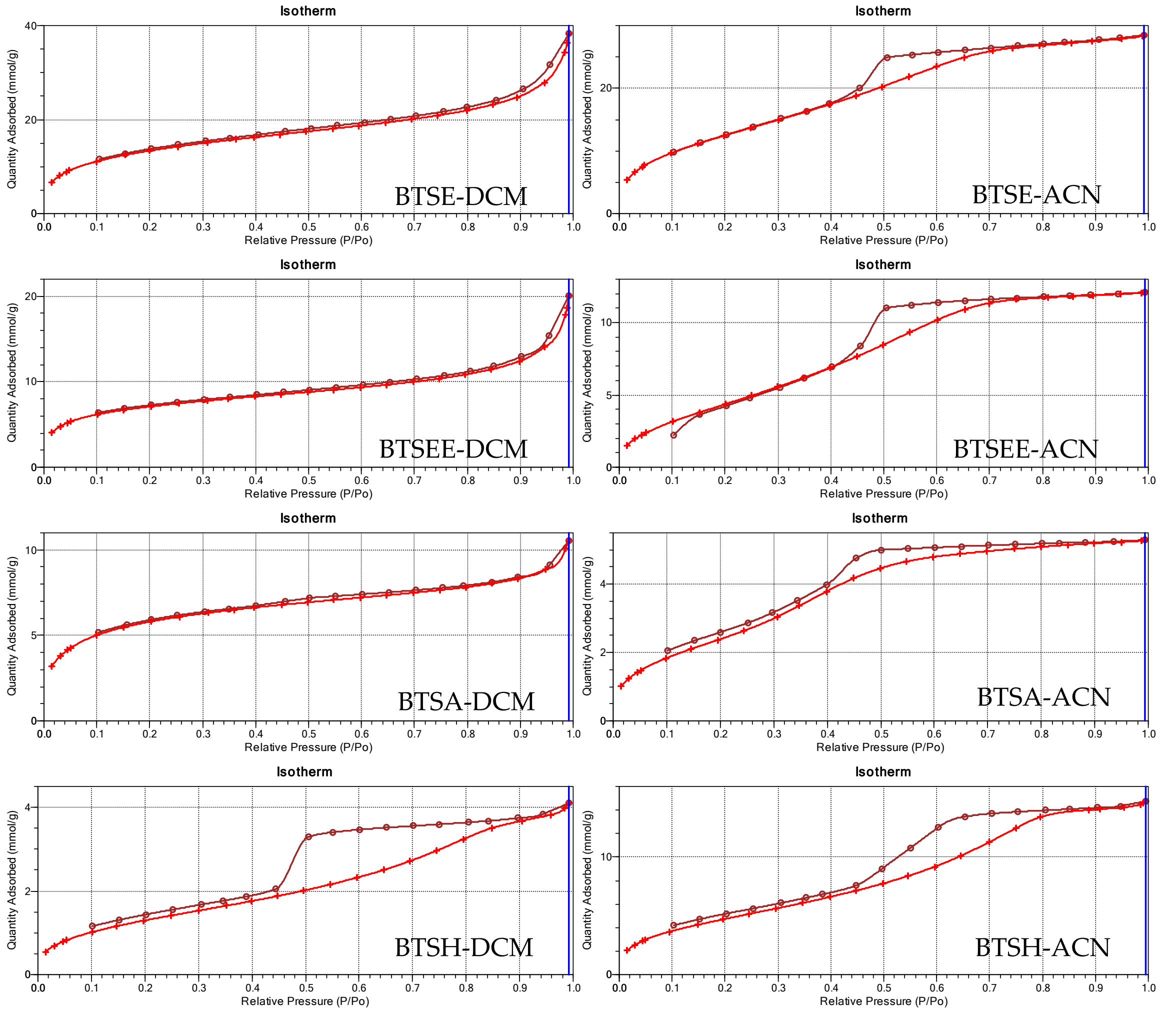
Isotherm
Quantity Adsorbed (mmol/g)
Relative Pressure (P/Po)
BTSE-DCM
BTSE-ACN
BTSEE-DCM
BTSEE-ACN
BTSA-DCM
BTSA-ACN
BTSH-DCM
BTSH-ACN

(a)

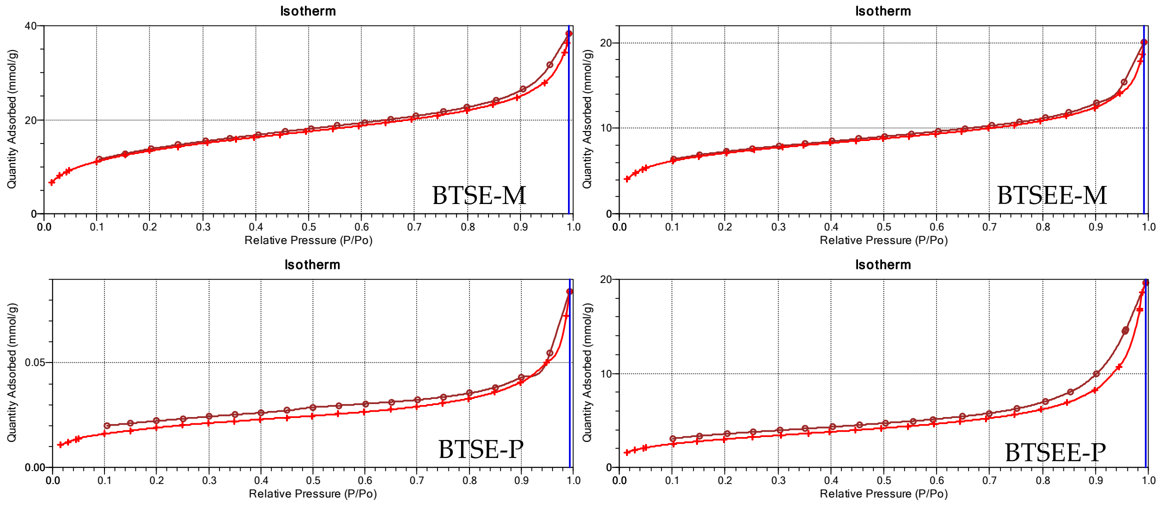
Isotherm
Quantity Adsorbed (mmol/g)
Relative Pressure (P/Po)
BTSE-M
BTSEE-M
BTSE-P
BTSEE-P

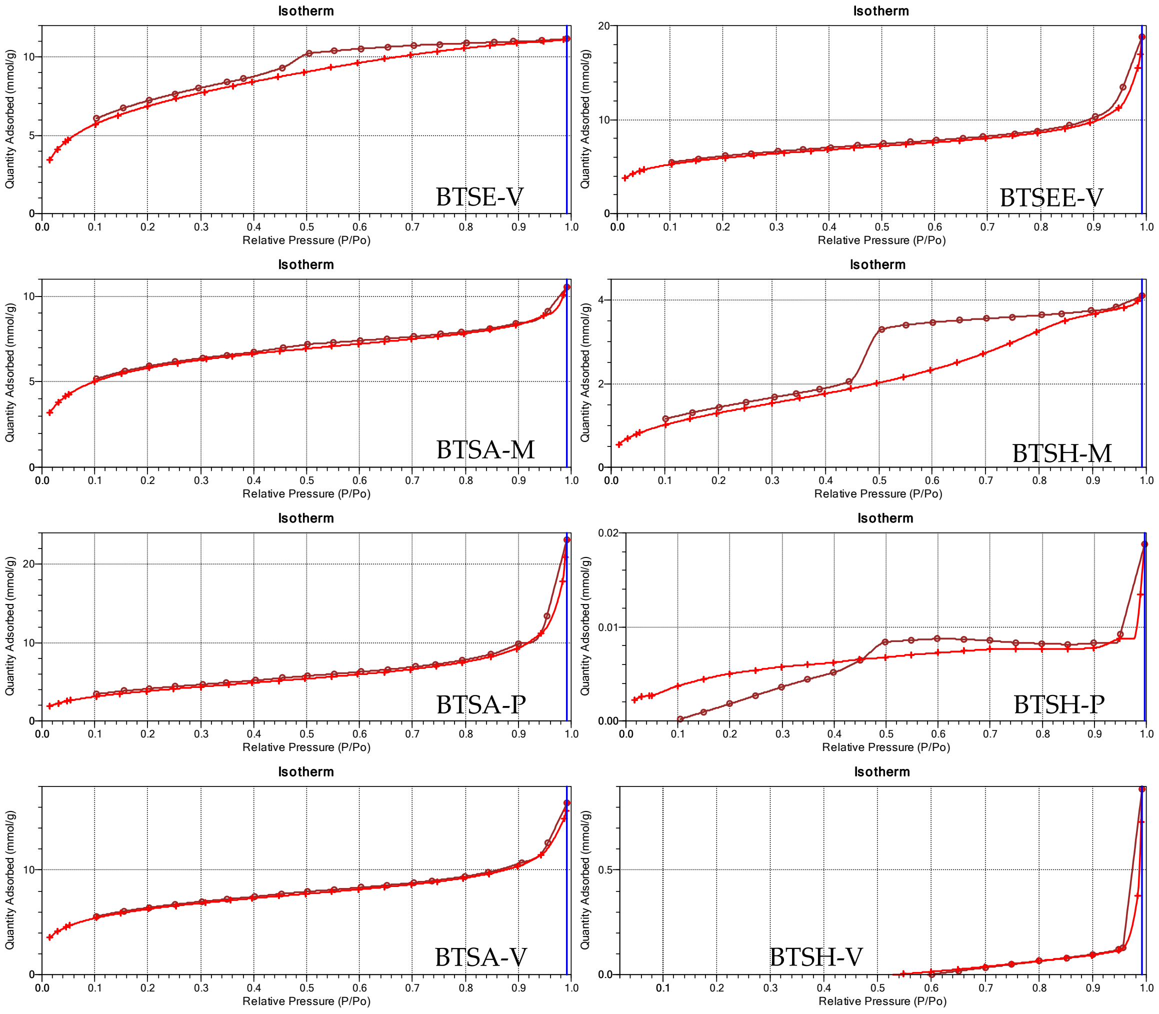

(b)

Figure S1. (**a**) Isotherm graphs from reactions with different cross-linkers in dichloromethane (DCM) and acetonitrile (ACN), Plus (light red) = adsorption, circle (dark red = desorption). (**b**) Isotherm graphs of materials from reactions with different corner-silanes and cross-linkers, P = phenyl, V = vinyl, M = methyl from DCM, Plus (light red) = adsorption, circle (dark red = desorption).

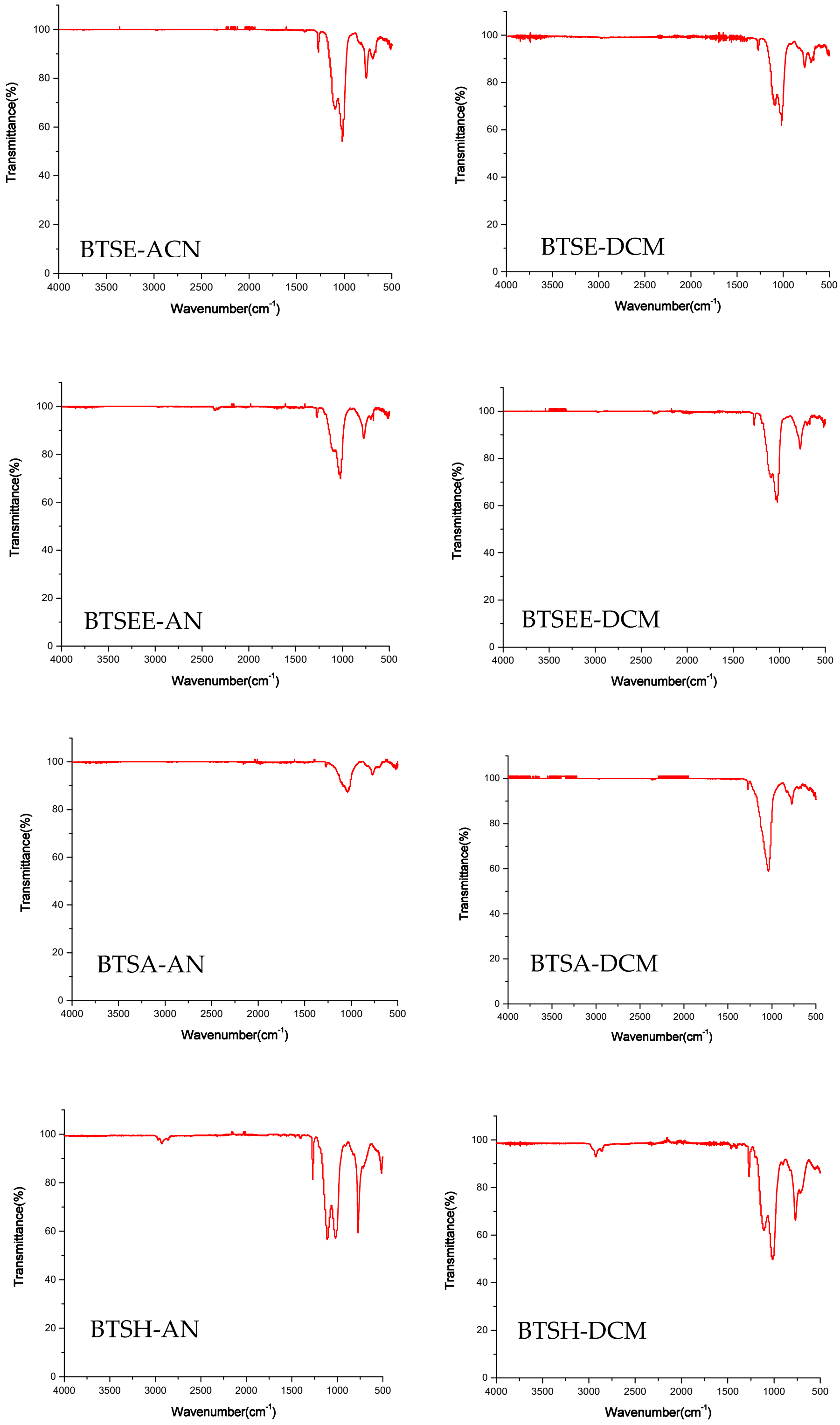

Figure S2. IR spectra of materials from reactions with different cross-linkers in dichloromethane (DCM) and acetonitrile (ACN).

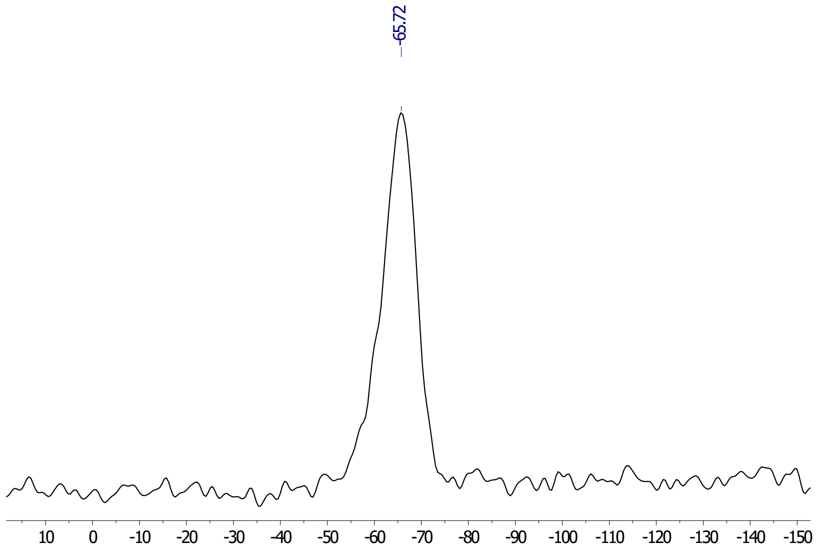

Figure S3. ^{29}Si NMR of methyl silsesquioxane based network polymer with hexyl spacer made in DCM. Note there are not extensive shoulders or side peaks indicating near complete reaction.

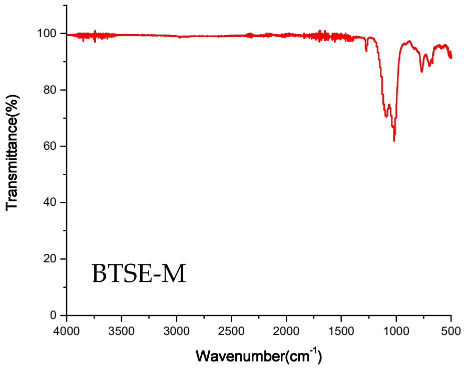
Transmittance(%)
Wavenumber(cm^{-1})
BTSE-M

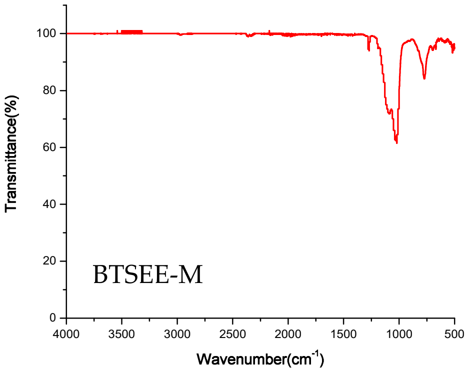
Transmittance(%)
Wavenumber(cm^{-1})
BTSEE-M

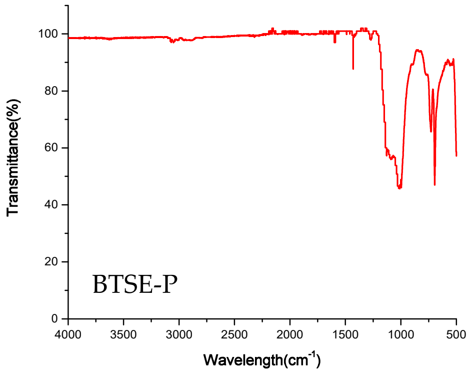
Transmittance(%)
Wavelength(cm^{-1})
BTSE-P

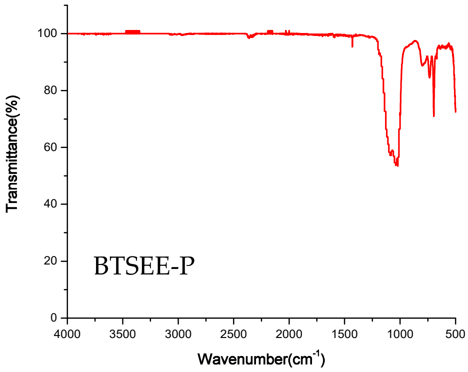
Transmittance(%)
Wavenumber(cm^{-1})
BTSEE-P

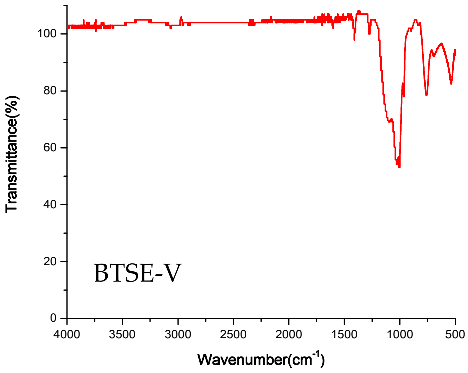
Transmittance(%)
Wavenumber(cm^{-1})
BTSE-V

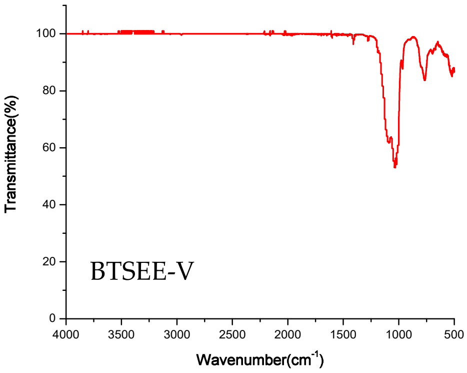
Transmittance(%)
Wavenumber(cm^{-1})
BTSEE-V

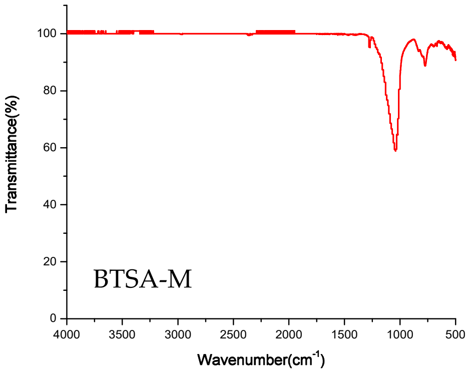
Transmittance(%)
Wavenumber(cm^{-1})
BTSA-M

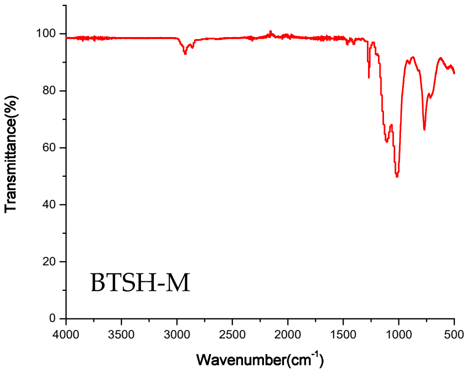
Transmittance(%)
Wavenumber(cm^{-1})
BTSH-M

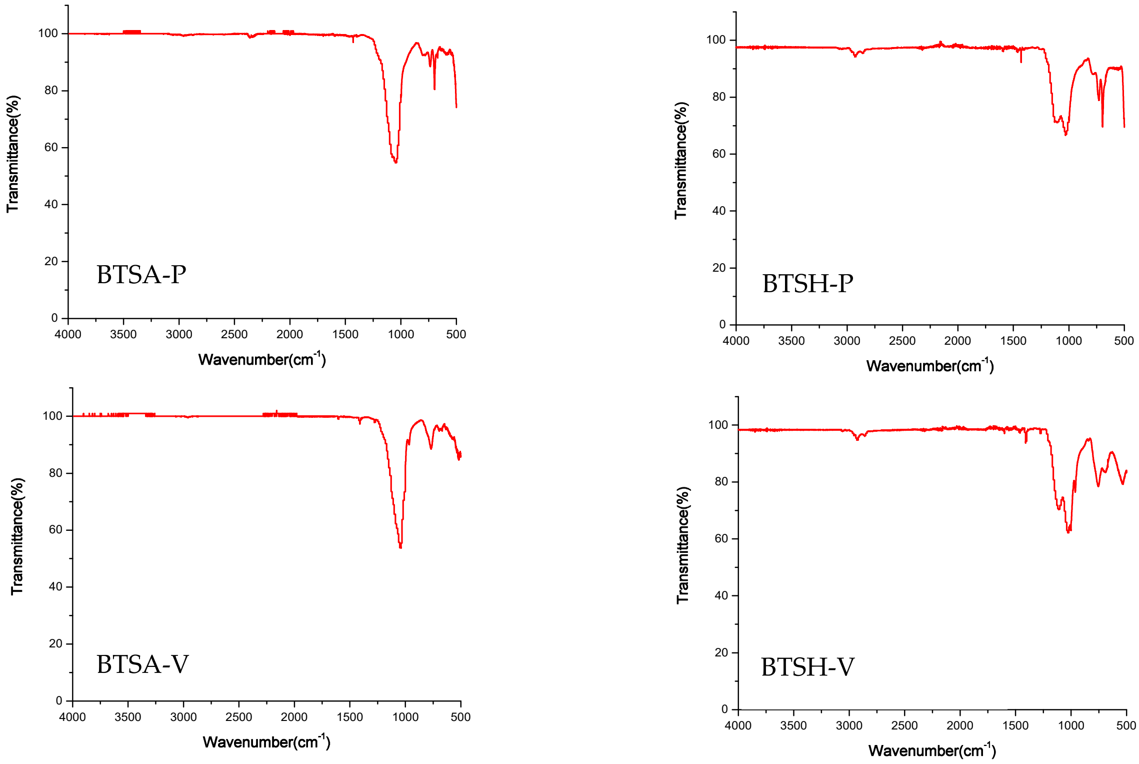

Figure S4. IR spectra of materials from reactions with different corner-silanes and cross-linkers, P = phenyl, V = vinyl, M = methyl from DCM.

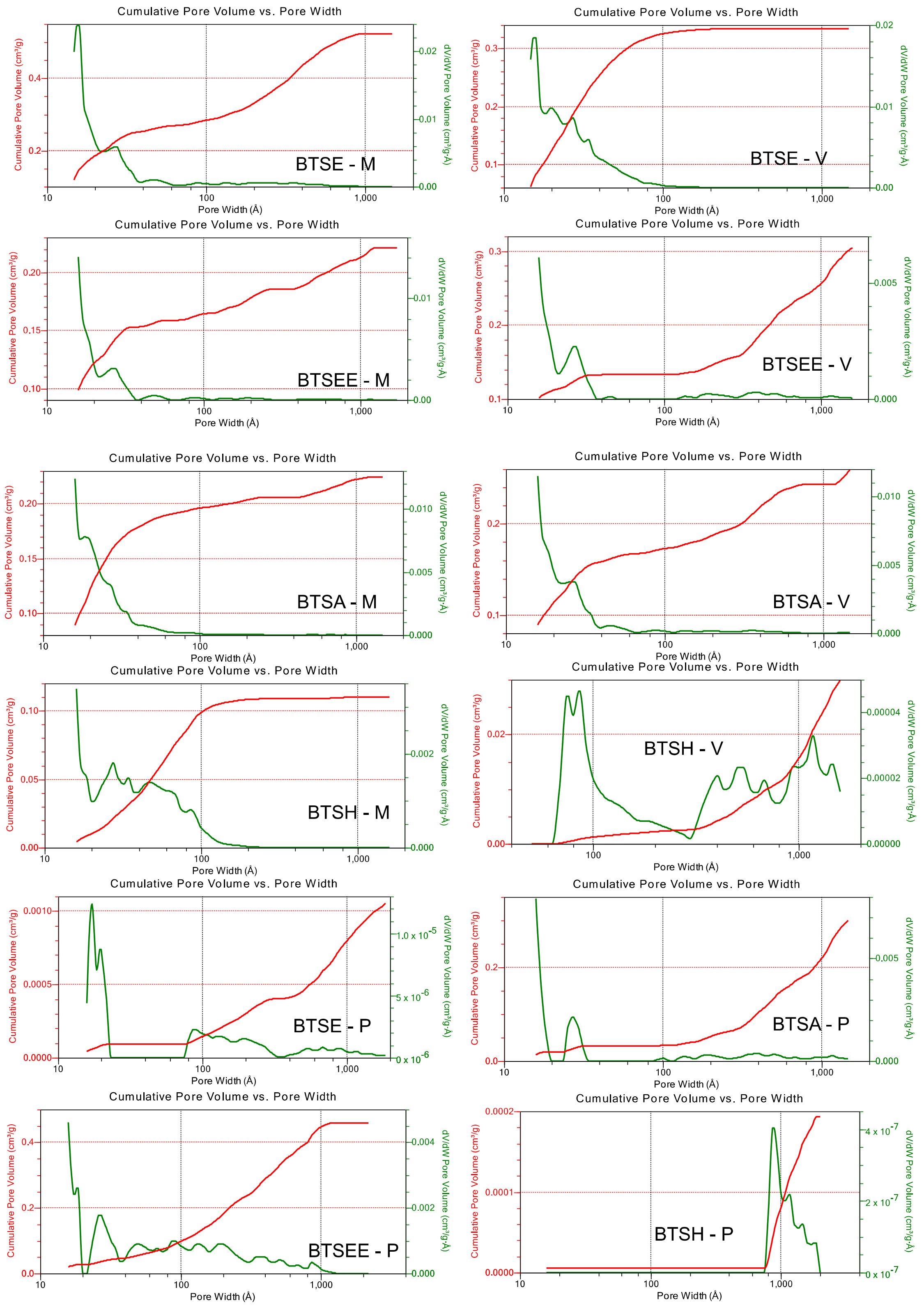

Figure S5. DFT pore size distribution plots and cumulative pore volume of materials from reactions with different corner-silanes and cross-linkers, P = phenyl, V = vinyl, M = methyl from DCM.
